# Increased Phosphaturia Accelerates The Decline in Renal Function: A Search for Mechanisms

**DOI:** 10.1038/s41598-018-32065-2

**Published:** 2018-09-12

**Authors:** Rafael Santamaría, Juan M. Díaz-Tocados, M. Victoria Pendón-Ruiz de Mier, Ana Robles, M. Dolores Salmerón-Rodríguez, Erena Ruiz, Noemi Vergara, Escolástico Aguilera-Tejero, Ana Raya, Rosa Ortega, Arnold Felsenfeld, Juan R. Muñoz-Castañeda, Alejandro Martín-Malo, Pedro Aljama, Mariano Rodríguez

**Affiliations:** 1 Nephrology Service, Reina Sofia University Hospital, Cordoba, Spain; 2 Maimonides Biomedical Research Institute of Cordoba (IMIBIC), Research Unit, Cordoba, Spain; 3 University of Cordoba, Cordoba, Spain; 4 Pathology Department, Reina Sofia University Hospital, Cordoba, Spain; 5 Nephrology Service, West Los Angeles VA, UCLA Los Angeles, Los Angeles, USA; 6 Red de Investigación Renal (RedInRen), Instituto de Salud Carlos III (ISCIII) and EUTOX, Madrid, Spain

## Abstract

In chronic kidney disease (CKD), high serum phosphate concentration is associated with cardiovascular disease and deterioration in renal function. In early CKD, the serum phosphate concentration is normal due to increased fractional excretion of phosphate. Our premise was that high phosphate intake even in patients with early CKD would result in an excessive load of phosphate causing tubular injury and accelerating renal function deterioration. In CKD 2–3 patients, we evaluated whether increased phosphaturia accelerates CKD progression. To have a uniform group of patients with early CKD, 95 patients with metabolic syndrome without overt proteinuria were followed for 2.7 ± 1.6 years. The median decline in eGFR was 0.50 ml/min/1.73 m^2^/year. Patients with a more rapid decrease in eGFR had greater phosphaturia. Moreover, the rate of decrease in eGFR inversely correlated with the degree of phosphaturia. Additionally, phosphaturia independently predicted renal function deterioration. In heminephrectomized rats, a high phosphate diet increased phosphaturia resulting in renal tubular damage associated with inflammation, oxidative stress and low klotho expression. Moreover, in rats with hyperphosphatemia and metabolic syndrome antioxidant treatment resulted in attenuation of renal lesions. In HEK-293 cells, high phosphate promoted oxidative stress while melatonin administration reduced ROS generation. Our findings suggest that phosphate loading in early CKD, results in renal damage and a more rapid decrease in renal function due to renal tubular injury.

## Introduction

It is generally accepted that in dialysis patients, high serum phosphate concentration is associated with cardiovascular disease and mortality^1,2^. Similarly, in patients with advanced chronic kidney disease (CKD), hyperphosphatemia predicts mortality and is associated with more rapid progression to end-stage renal disease^3^. Even in the general population, serum phosphate concentration in the high normal range predicts mortality^1–4^. Thus, in CKD stage 4 and 5 patients, there is general agreement that the elevated serum phosphate concentration should be reduced toward a normal level^5^. In CKD stages 2 and 3 patients, serum phosphate is not usually increased. However, it is uncertain whether increased phosphaturia resulting from high dietary phosphate intake has a deleterious effect on renal function^6^.

In CKD stages 3 to 5 patients, the magnitude of phosphaturia and the high serum phosphate levels have been reported to attenuate the anti-proteinuric effect of a very low protein diet^7^. However, an independent effect of increased phosphaturia on the progression of renal disease in early CKD patients has not been evaluated. Experimental work has shown that a high phosphate concentration produces tubular cell damage^8^, promotes apoptosis of proximal tubular cells and induces the expression of profibrotic and proinflammatory cytokines^9^.

In moderate CKD, the serum concentration of phosphate is maintained within normal levels because of an increase in fractional excretion of phosphate. In essence, total urinary excretion of phosphate should reflect intestinal absorption of phosphate^6^. Our premise was that even in the absence of hyperphosphatemia, a high phosphate intake in early CKD patients with their decreased number of nephrons would result in an excessive tubular load of phosphate, which in turn, would cause tubular injury and accelerate the deterioration in renal function. To date, this important clinical issue has not been properly studied in patients with early CKD.

In the present study, we evaluated whether the magnitude of phosphaturia was associated with the progression of CKD in a homogenous group of stages 2 and 3 CKD patients with metabolic syndrome without overt proteinuria. To evaluate potential mechanisms, additional studies were performed in rats to assess the changes in kidney structure, function and protein expression induced by excessive phosphaturia. Finally, a direct effect of a high phosphate concentration on kidney cells in culture was also studied.

## Results

### Patients

**Figure 1:**
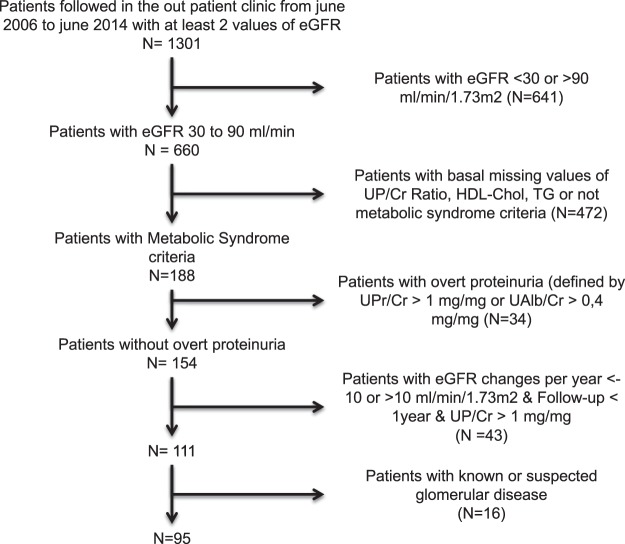
Flow chart showing details of how patients were selected. UP/Cr: Urinary Phosphate/creatinine; HDL-chol: High Density Lipoprotein Cholesterol; TG: Triglycerides, UPr/Cr: Urinary Protein/Creatinine; UAlb/Cr: Urinary Albumin/creatinine.

The flow chart for patient selection is summarized in Fig. 1. Of the patients included in the study, the median decline in eGFR was 0.5007 ml/min/1.73 m^2^ per year. Patients were classified as progressors if the decline in eGFR was greater than 0.5007 ml/min/1.73 m^2^/year and non-progressors when the decline in eGFR was less than 0.5007 ml/min/1.73 m^2^/year. Demographics and clinical characteristics of the study patients are shown in Table 1. Age, body mass index and abdominal circumference were similar in progressors and non-progressors. Control of blood pressure was also similar in both groups. Most patients required blood pressure medications and the type of medication prescribed, particularly renin angiotensin system inhibitors or blockers, was similar in progressors and non-progressors. However ischemic heart disease was more frequent among progressors than non-progressors. The use of anti-platelet aggregation therapy was similar in both groups. The frequency of diabetes, the prescription of oral hypoglycemic agents and the use of insulin were not different in progressors and non-progressors. Likewise, treatment of dyslipidemia was similar in both groups.

**Table 1:** Demographic and clinical characteristics of patients classified as progressors and non-progressors based on the decline in renal function.

	All (n = 95)	Non-Progressors (n = 48)	Progressors (n = 47)	P Value
Age (years)	63.1 ± 11.5	62.5 ± 11.7	63.7 ± 11.4	0.519
Gender				
Male	57 (60%)	29 (60.4%)	28 (59.6%)	0.933
Female	38 (40%)	19 (39.6%)	19 (40.4%)	
Abd. Circum (cm)	107.3 ± 9.4	105.4 ± 5.5	109.0 ± 12.1	0.466
BMI (kg/m2)	30.5 ± 4.0	30.5 ± 3.6	30.5 ± 4.4	0.655
Systolic BP (mmHg)	148 ± 23	145 ± 23	152 ± 24	0.200
Diastolic BP (mmHg)	85 ± 14	87 ± 15	85 ± 12	0.478
HTN (%)	97.9	97.9	97.9	0.988
T2DM (%)	40.0	35.4	44.7	0.357
OSA (%)	22.1	22.9	21.3	0.847
CHF (%)	9.5	10.4	8.5	0.751
CHD (%)	13.7	6.2	21.3	0.033
PA (%)	12.6	6.2	19.1	0.580
CVD (%)	9.5	10.4	8.5	0.751
ACE-i (%)	28.4	31.2	25.5	0.537
ARB (%)	70.5	64.6	76.6	0.199
Diuretics (%)	65.3	62.5	68.1	0.568
CCB (D) (%)	52.6	45.8	59.6	0.180
BB (%)	43.2	47.9	38.3	0.344
CAB (%)	33.7	33.3	34.0	0.942
Other antihyper drugs. (%)	12.6	16.7	8.5	0.232
OH (%)	25.3	18.8	31.9	0.140
Insulin (%)	17.9	20.8	14.9	0.450
Statins (%)	47.4	47.9	46.8	0.914
Fibrate/Omega3 (%)	17.9	16.7	19.1	0.752
Antiplatelet (%)	45.3	41.7	48.9	0.477

Mean ± SD.

Progressors (decline in eGFR >0.5 ml/min/1.73 m^2^/year) and non-progressors (decline in eGFR <0.5 ml/min/1.73 m^2^/year).

Abbreviations: Abd. Circum: Abdominal Circumference. BMI: Body Mass Index; HTN: Arterial Hypertension; T2DM: Type 2 Diabetes Mellitus; OSA: Obstructive Sleep Apnea Syndrome; CHF: Congestive Heart Failure; CHD: Coronary Heart Disease; PA: Peripheral Arteriosclerosis; CVD: Cerebrovascular Disease; ACE-i: Angiotensin Converting Enzyme Inhibitor; ARB: Angiotensin Receptor Blocker; CCB (D): Calcium Channels Blocker (Dihydropyridine); BB: Beta-blocker; CAB: Central Alfa-Blocker; OH: Oral Hypoglycemic Agent.

Biochemical parameters for serum and urine are shown in Tables 2 and 3. Serum phosphate levels were within normal limits in both progressors and non-progressors. There were no differences between the two groups in any serum value (Table 2). In urine, the values of phosphate/creatinine and urea/creatinine were increased in progressors as compared with non progressors (p = 0.012 and p = 0.025, respectively) (Table 3). The mean baseline eGFR was similar in both groups (Table 3). As defined by the study criteria, the rate of change in eGFR was less in non-progressors, 2.6 ± 2.9 ml/min/1.73 m^2^ per year than in the progressors, −2.8 ± 2.3 ml/min/1.73 m^2^ per year.

**Table 2:** Serum biochemical values.

	All (n = 95)	Non-Progressors (n = 48)	Progressors (n = 47)	P value
Glucose (mmol/L)	6.7 ± 2.65	6.8 ± 3.34	6.8 ± 1.71	0.164
Hb1Ac (%)	6.9 ± 1.46	6.9 ± 1.80	6.9 ± 0.96	0.288
Urea (mmol/L)	8.4 ± 3.41	8.3 ± 3.35	8.6 ± 3.50	0.602
Creatinine (μmol/L)	108 ± 35.1	108 ± 35.9	108 ± 34.6	0.979
Sodium (mmol/L)	141 ± 3.0	141 ± 3.5	141 ± 2.4	0.498
Bicarbonate (mmol/L)	25 ± 4.0	25 ± 4.1	25 ± 3.4	0.701
Potasium (mmol/L)	4.4 ± 0.64	4.4 ± 0.76	4.3 ± 0.51	0.548
Magnesium (mmol/L)	0.91 ± 0.121	0.92 ± 0.121	0.90 ± 0.122	0.238
Calcium (mmol/L)	2.44 ± 0.09	2.43 ± 0.09	2.45 ± 0.108	0.609
Phosphate (mmol/L)	1.09 ± 0.169	1.10 ± 0.172	1.09 ± 0.166	0.748
Uric Acid (μmol/L)	377 ± 103	390 ± 94.3	363 ± 110	0.156
Albumin (g/L)	4.5 ± 0.5	4.6 ± 0.48	4.5 ± 0.43	0.328
Triglycerides (mmol/L)	2.3 ± 1.37	2.3 ± 1.6	2.3 ± 1.17	0.772
HDL Cholesterol (mmol/L)	1.1 ± 0.30	1.0 ± 0.20	1.1 ± 0.38	0.495
LDL Cholesterol (mmol/L)	3.1 ± 0.87	3.1 ± 0.88	3.02 ± 0.87	0.555
Total Cholesterol (mmol/L)	5.1 ± 1.01	5.1 ± 1.05	5.06 ± 0.98	0.757
Hemoglobin (mmol/L)	8.5 ± 1.04	8.6 ± 1.02	8.4 ± 1.06	0.285
Ferritin (μg/L)	128 ± 177	147 ± 170	111 ± 183	0.065
C Reactive Protein (mmol/L)	53 ± 88.6	57 ± 109.4	50 ± 61.5	0.685

**Table 3:** Urine values and eGFR in Non-progressors and Progressors.

	All (n = 95)	Non-Progressors (n = 48)	Progressors (n = 47)	P value
Sodium/Creatinine ratio (mEq/mg)	1.3 ± 0.7	1.2 ± 0.8	1.3 ± 0.7	0.384
Potasium/Creatinine ratio (mEq/mg)	0.4 ± 0.2	0.5 ± 0.2	0.4 ± 0.2	0.578
Chloride/creatinine ratio (mEq/mg)	1.2 ± 0.7	1.1 ± 0.7	1.3 ± 0.8	0.239
Calcium/creatinine ratio (mg/mg)	0.1 ± 0.1	0.1 ± 0.1	0.1 ± 0.1	0.342
Protein/creatinine ratio (mg/mg)	0.14 ± 0.12	0.13 ± 0.12	0.14 ± 0.13	0.732
Albumin/creatinine ratio (mg/mg)	0.05 ± 0.08	0.04 ± 0.06	0.07 ± 0.1	0.139
Phosphate/creatinine ratio (mg/mg)	0.61 ± 0.18	0.57 ± 0.16	0.66 ± 0.18	0.012
Urea/creatinine (mg/mg)	16,8 ± 5,31	15,6 ± 4,74	18 ± 5,63	0.025
Baseline eGFR (ml/min/1.73 m^2^)	62 ± 19	62 ± 18	62 ± 20	0.988
Final eGFR (ml/min/1.73 m^2^)	62 ± 22	68 ± 20	55 ± 22	*0.004* ^*#*^
Follow-up (years)	2.7 ± 1.6	2.6 ± 1.5	2.8 ± 1.8	0.929
Rate of change in eGFR per year (ml/min/1.73 m^2^)	−0.5007 (−2.2–1.9)	2.6 ± 2.9	−2.8 ± 2.3	0

Mean ± SD. ^#^Different by definition because of division to non-progressors and progressors. Abbreviations: eGFR: Estimated Glomerular Filtration Rate; Hb1Ac: glycosylated hemoglobin.

The results of simple linear correlation analysis between the rate of decline in eGFR (dependent variable) and the continuous variables that in the comparison of progressors vs non-progressors with a P value < 0.2 in Tables 2 and 3, are shown in Table 4. The serum concentration of uric acid, systolic blood pressure and urinary urea/creatinine and albumin/creatinine (Alb/Cr) ratios did not show significant correlation. However, the decrease in eGFR did correlate significantly with P/Cr (r = −0.259, p = 0.011) suggesting that phosphaturia may negatively affect the eGFR.

**Table 4:** Simple Linear Correlation analysis.

Independent Variable	Correlation coefficient	Significance
Glucose	−0.110	0.290
Uric acid	0.189	0.068
Ferritin	0.140	0.187
Systolic BP	−0.190	0.078
Urinary Urea/Cr	−0.201	0.051
Urinary Alb/Cr	−0.187	0.070
Urinary P/Cr	−0.259	0.011

Dependent variable: Rate of change in eGFR.

eGFR: Estimated Glomerular Filtration Rate; BP: blood pressure; Cr: creatinine; Alb: albumin; P: phosphate.

Next a multiple linear regression analysis was performed using the change in eGFR as dependent variable and urine P/Cr as independent variable together with other variables that a priori were known to have the potential to accelerate the decline in eGFR: age, systolic blood pressure, low-density lipoprotein, uric acid, Alb/Cr ratio, ACE inhibitors-ARBs, glucose level, baseline CKD-EPI and ischemic heart disease. The results are shown in Table 5A. In addition to the P/Cr ratio in urine (p = 0.004), only systolic blood pressure (p = 0.01) and baseline eGFR (p = 0.011) had a significant effect on the change in eGFR. Finally, a mathematical model including the same variables that in the multiple regression model was generated using logistic regression to identify factors that could potentially predict the progression in renal function deterioration (progressors vs non-progressors) and only the urine P/Cr and Alb/Cr ratios were significant predictors of the change in eGFR (Table 5B).

**Table 5:** A. Forward stepwise Multiple Linear Regression, dependent variable: Change in estimated Glomerular Filtration Rate (eGFR) and B. Logistic Regression, dependent variable: Progressor status.

Multiple Linear Regression				
Independent Variable	Coefficient	Standard Error	Significance	
Constant	6.8	2.7	0.016	
Urine Phosphate/Creatinine (mg/mg)	−6.3	2.1	0.004	
Systolic Blood Pressure (mmHg)	−0.04	0.02	0.01	
Baseline eGFR (ml/min/1.73 m^2^)	0.05	0.02	0.011	
**Logistic Regression**				
**Independent Variable**	**Coefficient**	**Standard Error**	**Significance**	**Odds ratio Exp (B)**
Constant	−2.93	0.96	0.002	0.053
Urine Phosphate/Creatinine (mg/mg)	4.197	1.4	0.003	66.45
Urine Albumin/Creatinine (mg/mg)	6.6	3.1	0.033	735.04

**Figure 2:**
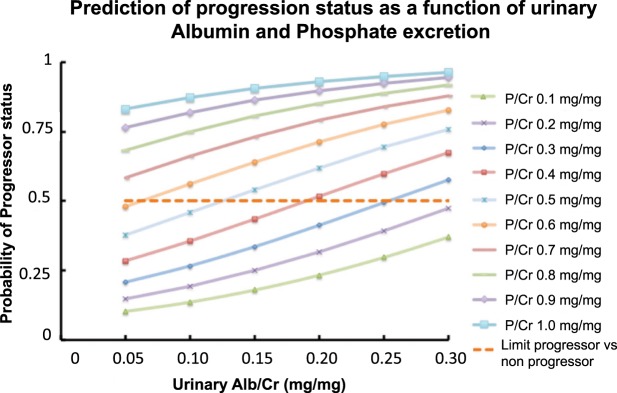
Prediction of progression status depending on urinary albumin/creatinine (Alb/Cr) and phosphate/creatinine (P/Cr) (mg/mg). P = 1/(1 + EXP (2.093 − 4.197xP/Cr − 6.6xAlb/Cr).

Prediction of progression status as a function of urinary P and albumin excretion is shown in Fig. 2. The curves were generated by the mathematical model derived from the logistic regression analysis presented in Table 5B. As shown in Fig. 2, each stratum of the urine P/Cr ratio was associated with a higher rate of eGFR progression and a higher Alb/Cr ratio further increased the rate of eGFR progression in each stratum of the P/Cr ratio.

### Animal studies with heminephrectomized rats

To study potential mechanisms for our clinical results, studies were performed in rats and with kidney cells. Plasma and urine biochemistry from sham and 1/2Nx rats on different phosphate diets is shown in Table 6. Serum phosphate and ionized calcium concentrations were similar in sham and 1/2 Nx rats in both the LPD and HPD groups. The plasma creatinine level was moderately increased in 1/2 Nx rats. The HPD was associated with an increase in the fractional excretion of phosphate in both the sham and 1/2 Nx groups. Plasma intact PTH levels were increased in rats fed a HPD as compared with LPD. The plasma concentration of intact FGF23 increased with the dietary content of phosphate in sham and 1/2 Nx rats and also was greater in the 1/2 Nx HPD rats than the sham HPD rats.

**Table 6:** Plasma and urine biochemical parameters.

	Sham LPD	Sham HPD	1/2Nx LPD	1/2Nx HPD
Plasma phosphate (mmol/L)	1.9 ± 0.2	2.1 ± 0.11	2.0 ± 0.13	2.1 ± 0.19
Plasma ionized calcium (mM)	1.3 ± 0.02	1.3 ± 0.01	1.3 ± 0.01	1.3 ± 0.02
Plasma creatinine (μmol/L)	44.2 ± 2.65	44.2 ± 0.88	61.8 ± 0.88^a,b^	61.8 ± 1.76^a,b^
FEPi (%)	0.4 ± 0.01	32.1 ± 7.49^a^	0.7 ± 0.08^b^	50.2 ± 7.03^a,c^
Plasma PTH (pg/ml)	21.9 ± 0.20	72.1 ± 7.66^a^	34.8 ± 4.26	65.9 ± 10.5^a,c^
Plasma FGF23 (pg/ml)	130 ± 33.9	272 ± 24.0^a^	119 ± 11.0^b^	454 ± 49.8^a,b,c^

^a^p < 0.05 vs Sham LPD; ^b^p < 0.05 vs Sham HPD; ^c^p < 0.05 vs 1/2Nx LPD.

LPD: Low Phosphate Diet; HPD: High Phosphate Diet.

FEPi: Fractional excretion of phosphate.

PTH: Parathyroid hormone.

FGF23: Fibroblast Growth Factor 23.

### Renal histology

**Figure 3:**
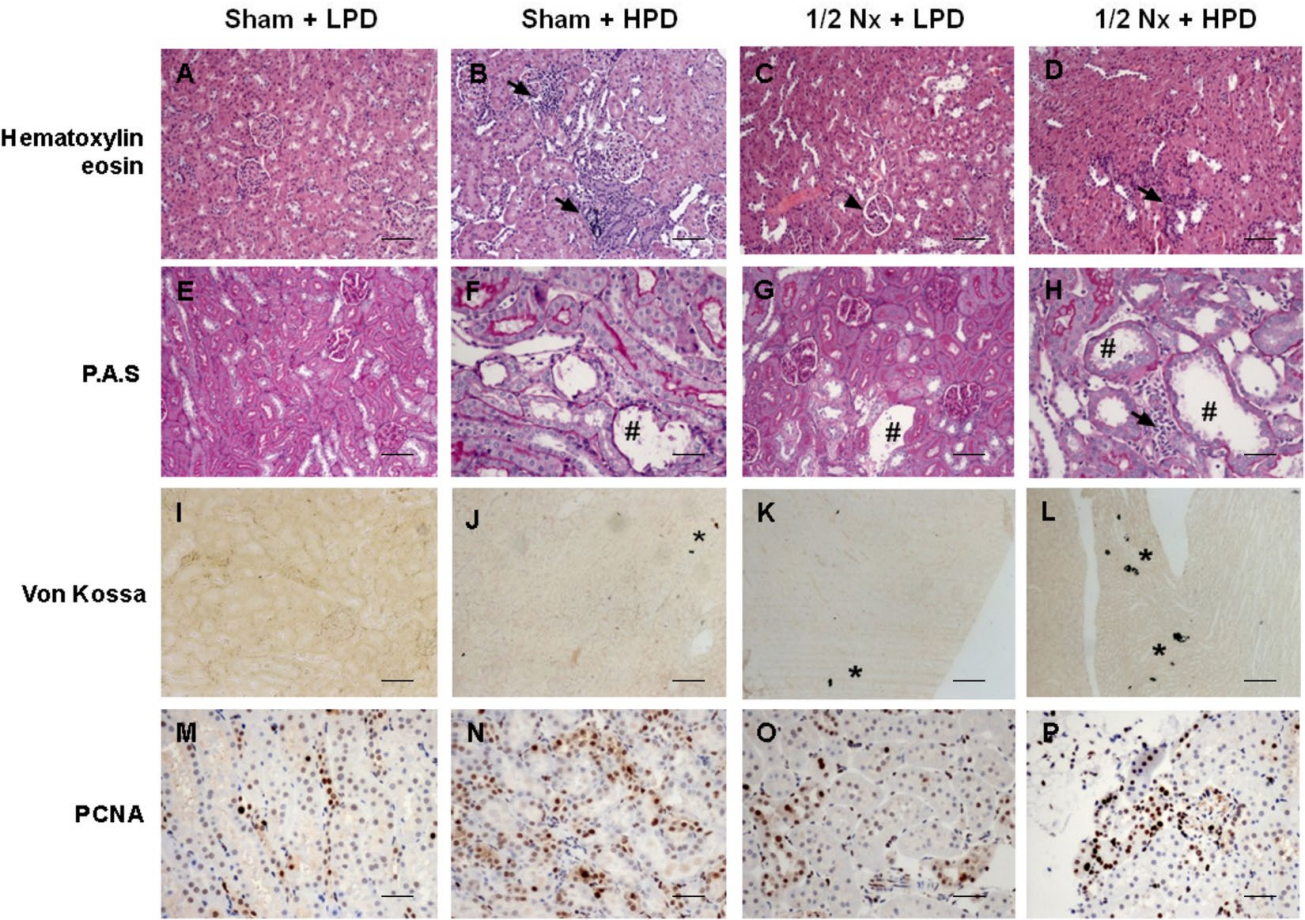
Excessive phosphaturia promotes renal injury. Renal samples from Sham + LPD, Sham HPD, 1/2Nx + LPD and 1/2Nx + HPD groups of rats were analyzed with hematoxilin-eosin staining (**A**–**D**), periodic acid staining (**E**–**H**), von Kossa (**I**–**L**) and PCNA immunostaining (**M**–**P**). Arrows: inflammatory reaction and hypercellularity; Arrowhead: glomerulus lobulation; ^#^Acute tubular necrosis; *presence of phosphate deposits. Magnification: x100. Scale Bar: 50 µm.

Microscopic evaluation of the kidney from sham rats on a HPD showed both renal tubular lesions and increased interstitial cellularity. These abnormalities were not observed in sham rats on a LPD. In the 1/2 Nx + LPD rats, lobulation of glomeruli with moderate mesangial hypercellularity was seen. The severity of tubular lesions and interstitial hypercellularity was accentuated in 1/2 Nx rats on a HPD (Fig. 3A–D). Moreover, in the two HPD groups, there was a loss of the integrity of the brush border membrane and even necrosis of proximal tubular cells with less epithelial cells and apoptotic bodies in the tubular lumen. Tubular cell toxicity, inflammatory reaction and hypercellularity were most prominent in the 1/2 Nx HPD rats (Fig. 3E–H). Von kossa staining documented the presence of several foci of calcification in kidneys from sham rats on a HPD. The calcification was much more evident in 1/2Nx rats on a HPD (Fig. 3I–L). PCNA immunohistochemical staining illustrated enhanced tubular cells proliferation in an attempt to replace damaged cells. While sham rats on a HPD had a significant increase in cell proliferation, PCNA staining with a parallel increase in inflammatory cell infiltrate was most evident observed in 1/2 Nx rats on a HPD (Fig. 3M–P).

### Renal expression of p65 and α-Klotho

**Figure 4:**
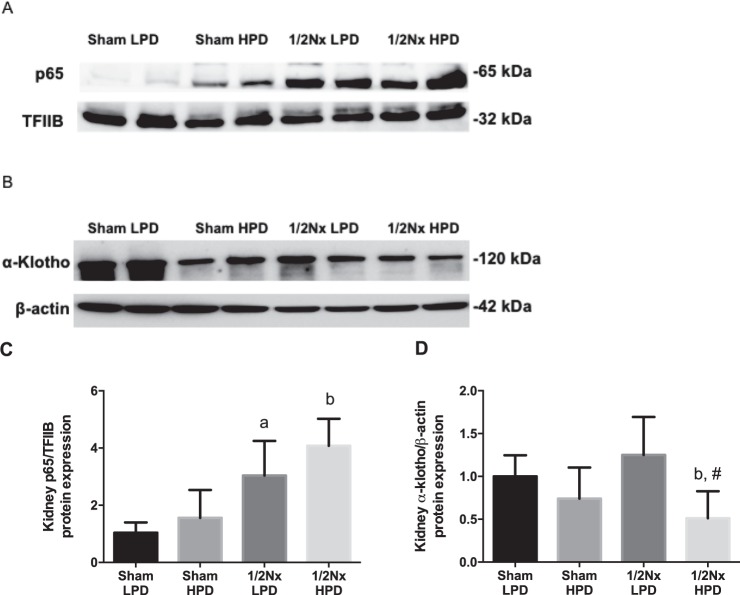
Burden phosphate reduces Klotho and promotes inflammation. Western blotting of protein extracts from kidney tissue. For detection of α-klotho (**A**) was used the cytoplasmic fraction and β-actin as loading control. For p65 fragment from NF-kB (**B**) detection was used the nuclear fraction of renal protein extracts and TFIIB as loading control. Quantification of western blots of Klotho (**C**) and p65 (**D**) were performed by measurement of average relative density and normalized to β-actin levels. ^a^p < 0.05 vs Sham LPD; ^b^p < 0.05 vs. Sham HPD and ^#^p < 0.05 vs. Nx1/2 LPD.

There was a tendency to increase the nuclear expression of p65 (inducible fragment of NFkB, a pro-inflammatory transcription factor) in rats with increased phosphaturia (Fig. 4A). These differences were significant in Nx1/2 rats as compared to their respective Sham groups (Fig. 4C). Renal Klotho expression was also evaluated by western blot and the results are shown in Fig. 4B and D. In rats on a HPD with the accompanying increase in phosphaturia, there was a decrease of renal Klotho that was remarkable in 1/2 Nx rats on the HPD as compared to Sham HPD (^b^p < 0.05) or Nx1/2 LPD (^#^p < 0.05).

### Oxidative stress

**Figure 5:**
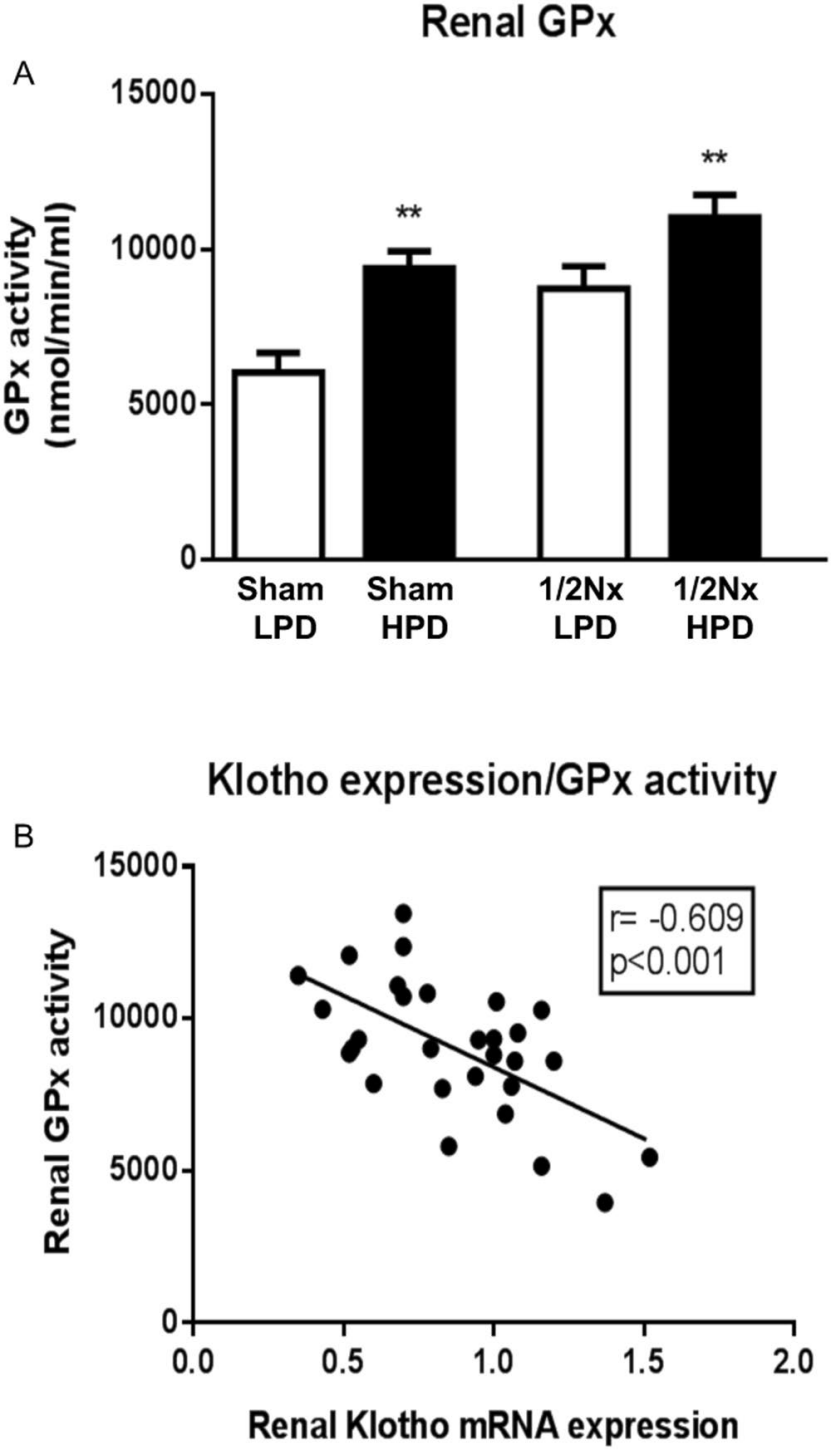
Elevated phosphaturia generates renal oxidative stress. Glutathione peroxidase (GPx) activity in renal tissue. (**A**) GPx activity was determined in 20 µg of total protein extracts of kidney samples by a coupled reaction with glutathione reductase. Units are expressed as nanomoles of NADPH oxidized per minute and milliliter. White bars are LPD (0.2%P diet) groups and black bars are HPD (1.2%P diet) groups. t-test analysis *p < 0.05, **p < 0.01. (**B**) Negative correlation between renal mRNA α-klotho expression and GPx activity.

Kidneys from rats fed a HPD, as compared to their sham or 1/2 Nx LPD controls, had increased activity of the critical antioxidant enzyme glutathione perioxidase (GPx) (Fig. 5A) suggesting that an increased renal load of phosphate may trigger an antioxidant response through an increased production of reactive oxygen species (ROS). Of interest was the finding of a significant negative correlation between GPx activity and renal α-Klotho expression (Fig. 5B).

### Animal studies with Zucker rats and antioxidant

**Figure 6:**
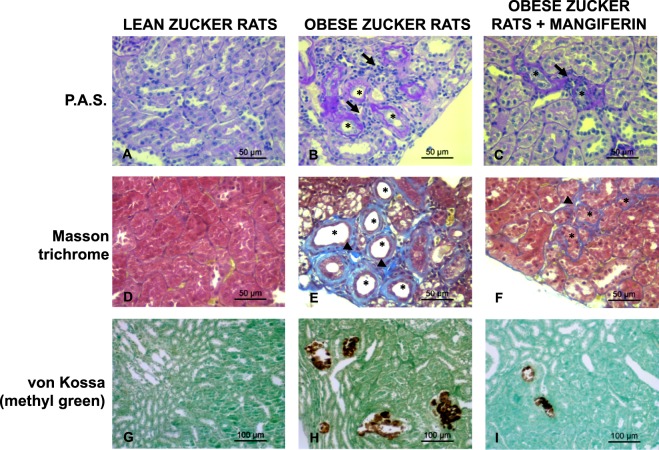
Treatment with Mangiferin reduces renal injury in obese Zucker rats. Kidney tissue samples from Lean, Obese Zucker rats and Obese Zucker rats plus 8 weeks of mangiferin treatment were analyzed with PAS stained sections (**A–C**) where tubular atrophy (asterisks) and peritubular inflammatory infiltrate (arrows) were observed. The thickening of the basement membrane was also evidenced in atrophic tubules (asterisks) with the Masson Trichrome, as well as interstitial fibrosis (arrowheads) (**D–F**). Mineral deposits are depicted by the brown color in von Kossa stain (**G–I**). Magnification: x400, scale Bar: 50 µm for PAS and Masson Trichrome, x200 and scale bar 100 µm for von Kossa.

The role of oxidative stress in the progression of renal injury was evaluated in Zucker rats, a model of metabolic syndrome, by treatment with the antioxidant Mangiferin or placebo during 8 weeks. Table 7, shows that at 16 weeks of age Zucker rats developed metabolic syndrome and dietary phosphate overload (secondary to increased food intake). Renal histology is shown in Fig. 6, control lean animals show normal histology; kidneys from obese Zucker rats receiving placebo presented tubulointerstitial lesions including focal areas of tubular atrophy with dilation, tubular hyperplasia and thickening of the tubular basement membrane, a moderate degree of interstitial fibrosis and inflammatory infiltrate surrounding the damaged tubules, with minor calcification. Antioxidant treatment with Mangiferin resulted in attenuation of renal lesions, mainly manifested by a reduction in tubular damage and interstitial fibrosis; the improvement of inflammatory infiltrate and the calcification was less remarkable (Fig. 6).

**Table 7:** Metabolic Syndrome and renal parameters in Obese rats treated or not with antioxidant.

	Lean-Zucker	Obese Zucker	Obese Zucker + Mangiferin
Weight Gain	59.7	255.3^a^	246,8^a^
Intake (g/day)	15.1 ± 1.62	26.6 ± 1.01^a^	25.4 ± 1.04^a^
Serum P, mg/dl	4.5 ± 0.83	6.9 ± 1.25^a^	7.4 ± 0.88^a^
Serum Creat, mg/dl	0.49 ± 0.083	0.44 ± 0.052	0.35 ± 0.053
Glucose, mg/dl	147 ± 11,7	184 ± 38,3	187 ± 21.2
Cholesterol, mg/dl	61 ± 6.1	113 ± 9.1^a^	105 ± 10.2^a^
LDL, mg/dl	4.4 ± 1.22	10.1 ± 3.89^a^	7.0 ± 3.97^a^
HDL, mg/dl	25.8 ± 2.86	42.6 ± 2.67	46.7 ± 2.56
Triglycerides, mg/dl	49 ± 10	709 ± 254.5^a^	841 ± 265.9^a^
Insulin, ng/ml	0.36 ± 0,12	11.7 ± 7.95^a^	10.6 ± 3.44^a^

^a^Significant differences at least p < 0.05 vs Lean Rats.

### *In vitro* studies with HEK-293

**Figure 7:**
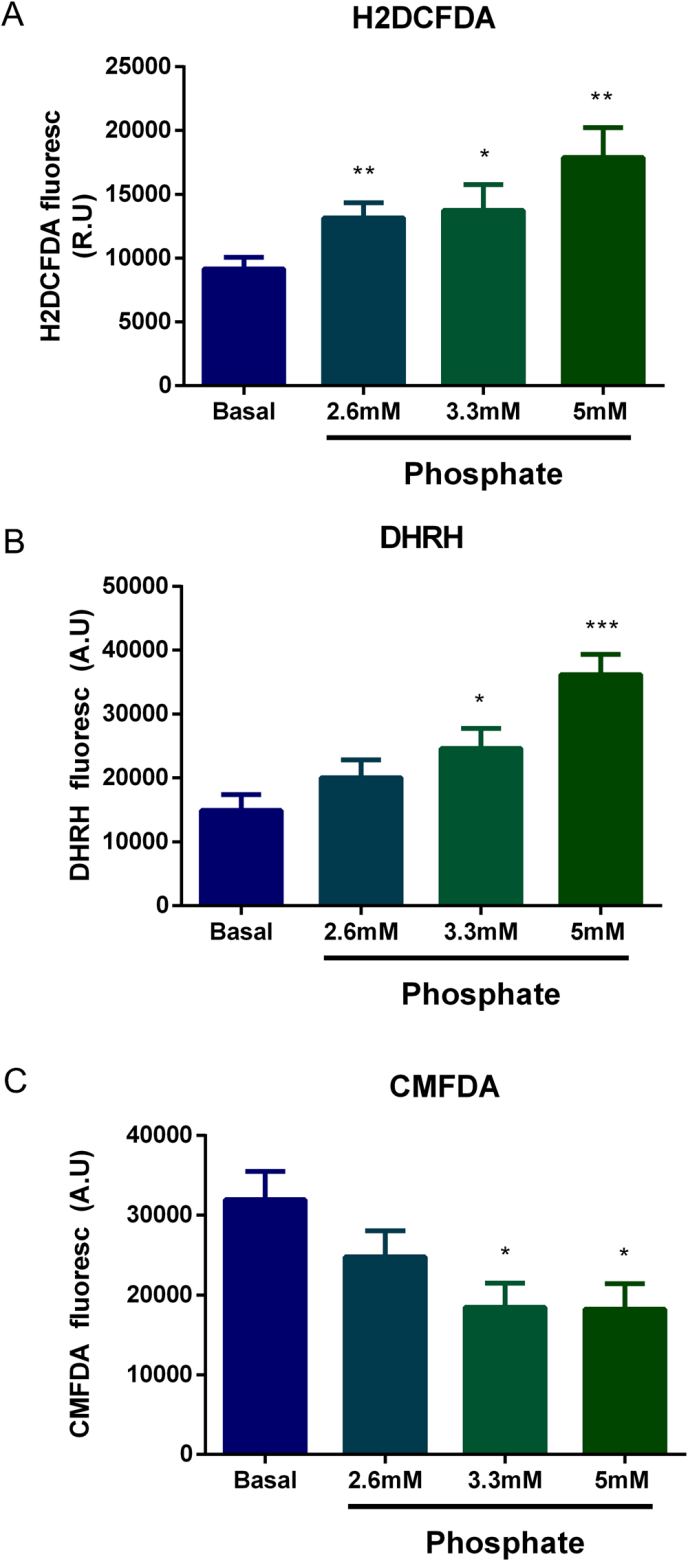
Phosphate increases Reactive Oxygen Species (ROS) in HEK-293 cells. Intracellular ROS was determined using the probes (**A**) H2DCFDA (detects presence of ROS unspecifically) (**B**) DHRH (determinates peroxynitrate) (**C**) CMFDA (detects levels of glutathione) after 24 hours of incubation with different concentrations of inorganic phosphate. (t-test analysis. *p < 0.05 vs basal phosphate; **p < 0.01 vs basal phosphate; ***p < 0.001 vs basal phosphate. Basal phosphate is the content of phosphate in the culture medium at 0.9 mM.

A direct effect of high phosphate concentration on the production of ROS by kidney cells was evaluated *in vitro*. HEK-293 cells were cultured in increasing concentrations of inorganic phosphate: basal (0.9 mM), 2.6, 3.3 and 5 mM. After 24 hours, the H2DCFA fluorescent probe showed the presence of ROS that increased in a phosphate concentration-dependent manner (Fig. 7A). Likewise, high phosphate concentration also increased endogenous production of peroxynitrites in HEK-293 cells as indicated by the higher fluorescence of the DHRH probe (Fig. 7B). Finally, high phosphate concentrations decreased the content of reduced glutathione reported by the probe CMFDA (Fig. 7C).

**Figure 8:**
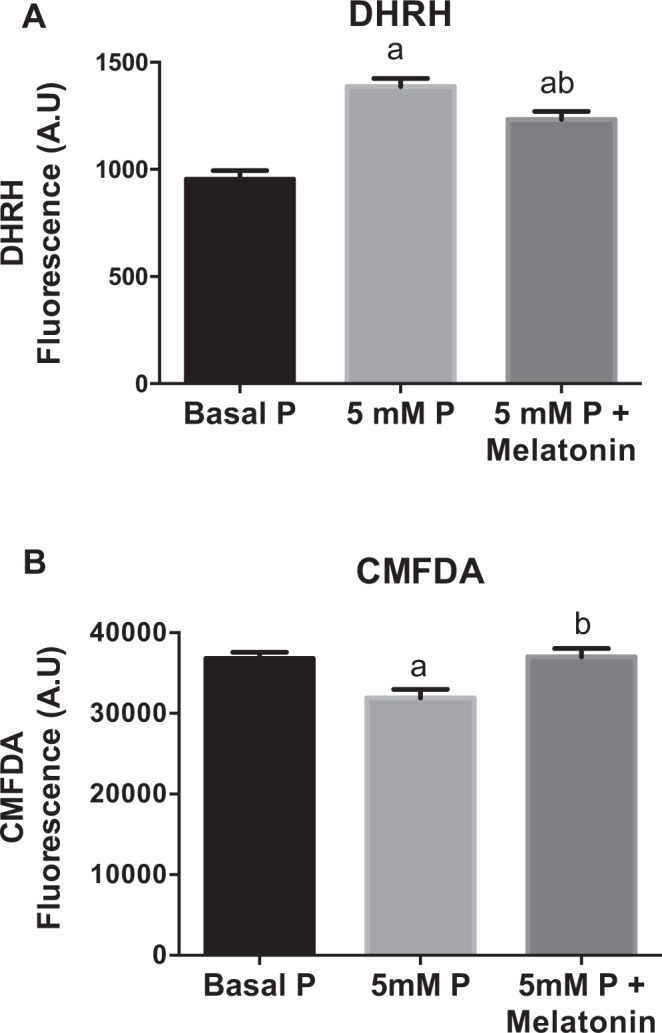
In HEK-293 cells, the addition of melatonin prevents oxidative stress induced by high levels of inorganic phosphate. Intracellular ROS were determined by using the probes (**A**) DHRH and (**B**) CMFDA after 24 hours of incubation in a medium containing normal P concentration (0.9 mM), high levels of phosphate (5 mM P) and high phosphate plus melatonin (10^−5^ M). ^a^p < 0.001 vs normal phosphate; ^b^p < 0.001 vs 5 mM phosphate.

Figure 8 shows that preincubation of HEK-293 cells with melatonin prevents the increase of DHRH intensity induced by high P concentration (5 mM) and also increases the content of glutathione (CMDFA) (p < 0.001).

## Discussion

The present study was performed to determine whether the progression of renal disease in patients with moderate renal failure and normal serum phosphate was affected by the amount of phosphaturia. After a mean follow up of almost 3 years, the results showed that phosphaturia predicted progression of renal disease. Our clinical results suggested that an excessive tubular load of phosphate may damage the kidney. This concept was validated by additional studies in experimental animals and cultured kidney tubular cells.

We selected a group of patients with metabolic syndrome without overt proteinuria to have a uniform group of patients with early CKD (stages 2 and 3). Because albuminuria is associated with CKD progression, the presence of overt proteinuria could have obscured the finding of an independent effect of phosphaturia on progression of renal disease. Although unexpected, it was interesting to observe that even a minor degree of albuminuria negatively affected renal function.

A strength of this work is that during the entire study, patients received their medical care from the same physician. Thus, there were uniform criteria for the use of medications and for the management of hypertension, dyslipidemia and glucose control. In addition, because metabolic syndrome is so common our findings from the present study can be extrapolated to a large number of patients.

The animal studies were designed to determine the site of the phosphaturia-induced kidney damage that was observed in patients. The aim was to explore whether kidney injury can be induced by increased phosphaturia without hyperphosphatemia. Histological analysis revealed that a HPD, predominantly in moderate renal failure (1/2Nx) rats, produced tubular cell toxicity, inflammatory infiltrates, and cell proliferation with the latter presumably to repair damage. There was also evidence of focal calcification although the inflammatory response was not circumscribed to the small area of calcification. The increased expression of p65 in kidneys from rats on a HPD indicates that excessive phosphate promotes inflammation. Furthermore, the increased activity of the antioxidant enzyme GPx, suggests a tissue response to oxidative stress in rats on a HPD. The influence of oxidative stress on renal damage was further demonstrated by studies in obese Zucker rats with metabolic syndrome. These animals showed hyperphosphatemia and significant renal lesions, as compared with their lean counterparts. These lesions were more evident at tubular level and were improved after antioxidant treatment. A direct effect of high phosphate concentration on oxidative stress was also shown in HEK-293 cells in which increased production of ROS was seen in response to a graded increase in extracellular phosphate concentration. This pro-oxidant effect of high P on HEK-293 cells was also supported by the *in vitro* studies showing a beneficial antioxidant effect of Melatonin. Finally, gene and protein expression of renal α- Klotho was reduced in rats on a HPD. Furthermore, the decrease in α- Klotho expression was proportional to the increase in GPx.

Our results are unable to disclose if the generation of oxidative stress and NF-kB activation are independent processes or they are tightly interrelated to each other. It is clear that in an early stage of kidney disease elevated phosphaturia promotes oxidative stress, p65 activation and renal injury. In previous work from our group it was observed that, in vascular smooth muscle cells treated with high phosphate and calcitriol, antioxidant treatment decreased p65 activation suggesting a central role of oxidative stress in p65 activation^7^.

Taken together one could assume that increased phosphaturia generates kidney injury through oxidative stress and inflammation that leads to a reduction in α-Klotho expression. Moreover, we have also recently demonstrated in animal models that excessive phosphaturia through Wnt-β-catenin activation, decreases renal Klotho expression which in turn, may prevent the phosphaturic action of FGF23^8^. Other authors have shown that high phosphate concentration is toxic and pro-apoptotic to tubular cells^9–11^. Of relevance, a recent work by Zhou *et al*.^12^ has shown that injured renal tubular cells stimulate renal fibrosis through activation of the fibroblast Wnt signalling system, a mechanism by which excessive phosphaturia may produce renal damage. Thus, these cited studies support our findings that phosphaturia has the potential to produce renal injury and accelerate CKD progression even in early stages of renal disease.

Based on our clinical and animal studies, it would appear that high phosphate intake increases the phosphate burden and the resulting phosphaturia produces secondary kidney damage by inducing tubular injury and interstitial fibrosis. Other studies have found that subjects with high serum phosphate had a higher risk for developing kidney disease^13–15^. In patients with more advanced renal disease, the progression of renal disease may be predetermined by other factors making it difficult to isolate phosphaturia as an independent factor predicting progression of renal disease. Moreover, in advanced CKD, the fractional excretion of phosphate is already very high limiting the capacity to further enhance phosphaturia. That is the reason hyperphosphatemia develops. At present, there are not studies in patients with CKD stages 2 to 3 in which the relationship between phosphaturia and progression of renal disease has been studied. In studies in patients with more advanced CKD from a variety of renal diseases and with different magnitudes of proteinuria, an effect of phosphaturia on renal disease progression has not been shown, but the limitation to further enhance phosphaturia may play a role^15,16^. Isakova T *et al*. found in patients with relatively preserved kidney function, GFR between 30 and 44 mL/min/1.73 m^2^, that high FGF23 was associated with significantly higher risk of end-stage renal disease. Unlike FGF23, neither PTH nor FePi were associated with mortality in fully adjusted models that excluded FGF23^16^. As compared with our patients, patients from the Isakova’s study had more advanced stage of CKD with higher levels of FGF23 and more severe cardiovascular disease. At this stage of kidney failure other factors besides proteinuria and FePi may play a more relevant role in the renal disease progression.

Efforts to reduce progression of renal disease may have significant social and economic impact. The mechanisms involved in kidney injury in CKD are multifactorial; an increased acid excretion per remaining nephron has been shown to produces interstitial fibrosis and metabolic acidosis due to CKD is associated with progressive deterioration of kidney function experimental animals^17^.

The results of the present study may serve as the basis for future interventional studies with dietary phosphate restriction or phosphate binders that are designed to determine if a reduction in intestinal absorption of phosphate in stages 2 to 3 CKD helps to preserve renal function. Moreover, besides the phosphate in food as protein, a significant burden of dietary phosphate is due to unrestricted intestinal absorption of phosphate salts present in beverages and food additives^18,19^. If an excess of phosphate absorption from food additives and beverages is shown to have negative effects on patients with early to moderate CKD, health authorities should be aware.

In conclusion, in patients with metabolic syndrome and early CKD, phosphaturia and only marginal albuminuria were associated with a deterioration in renal function. Experimental studies showed that excessive phosphaturia was associated with renal injury, inflammation, oxidative stress and decreases in renal Klotho.

## Methods

### Study design in patients

This is an observational retrospective study in patients followed in our renal outpatient clinic between June 2006 and June 2014. The study was approved by the Reina Sofia Hospital Ethics Committee and an informed consent was obtained. Authors state our adherence to the Declaration of Helsinki.

All studied patients had metabolic syndrome and had to fulfill three of the following five criteria to be considered metabolic syndrome^20^: (1) Waist circumference ≥88 cm in women and ≥102 cm in men; (2) serum triglycerides ≥1.65 mmol/L or drug treatment for hypertriglyceridemia; (3) low high-density lipoprotein, men <1 mmol/L, women <1.25 mmol/L or drug treatment; (4) elevated fasting glucose (≥6.05 mmol/L) or drug treatment for diabetes mellitus; and (5) systolic blood pressure ≥130 and/or diastolic blood pressure ≥85 mmHg or treatment for arterial hypertension. Glomerular, tubular and interstitial pathologies were excluded.

Patients were 18 to 86 years old with an estimated glomerular filtration rate (eGFR) between 90 and 30 ml/min/1.73 m^2^. Only patients with minimal proteinuria (albumin/creatinine ratio <0.4 mg/mg and protein/creatinine ratio <1 mg/mg) were included. Patients were closely followed as outpatients during a period that ranged from 1 to 6 years. They were seen in clinics at least twice per year. On each visit, the same physician adjusted patient medications and blood and urine were collected for laboratory tests.

Baseline data obtained from the patient medical record included age, sex, body mass index, waist circumference, blood pressure, and pre-existing cardiovascular disease. Laboratory tests included serum creatinine, glucose, calcium, phosphate, magnesium, albumin, and lipids and urine phosphate, protein, albumin and creatinine. Medications including angiotensin-converting enzyme (ACE) inhibitors, angiotensin receptor blockers (ARB), loop and thiazide diuretics, statins, fibrates, xanthine oxidase inhibitors, oral hypoglycemic drugs and insulin use, and anticoagulation and antiplatelet drugs were recorded. Hypertension was defined as a systolic blood pressure >140 mmHg and/or diastolic blood pressure >90 mmHg or a previous diagnosis of hypertension on blood pressure medication. Pre-existing cardiovascular disease was defined as a clinical history of coronary artery disease, hypertensive heart disease, valvular heart disease, cardiomyopathy, arrhythmia, peripheral vascular disease, cerebral infarction, cerebral hemorrhage, or subarachnoid hemorrhage. Abnormalities in glucose metabolism were defined as a fasting glucose level >6.05 mmol/L or the use of hypoglycemic medication.

Blood was collected for measurement of standard serum biochemistry and hemogram. Morning urine samples were used for quantification of urine electrolytes, phosphate, albumin, protein and creatinine. Serum albumin, serum and urinary levels of creatinine, urea, sodium, phosphate, potassium and concentration of albumin and protein in urine were measured with an Architect c-16000 (Abbott®, Chicago, Illinois (USA)). The eGFR was calculated by CKD-EPI formula^21^. Hemogram was measured by Pentra 120 Retic® (ABX, France).

Daily habits and medications for control of blood pressure, serum lipids and glucose were maintained and adjusted according to values obtained on each visit. Non-steroidal anti-inflammatory drugs were avoided.

The rate of change in eGFR (ml/min per 1.73 m^2^ per year) was calculated from the difference between last visit and baseline. This change in eGFR coincided with the calculated, using data points (range 2 to 8 points) throughout the follow up period with the use of the least-squares method.

### Animal Models

Male Wistar rats underwent right nephrectomy to obtain a heminephrectomy (1/2Nx) and controls animals underwent sham operation. The animals were fed either a low phosphate (0.2%) diet (LPD) or high phosphate (1.2%) diet (HPD). Rats were allocated to the following groups: Sham + LPD (phosphate 0.2%, calcium 0.6%), n = 9; Sham + HPD (phosphate 1.2%, calcium 0.6%), n = 8; 1/2 Nx + LPD, n = 12 or 1/2 Nx + HPD, n = 12. After three weeks, rats were euthanized and exsanguinated by aortic puncture. The left kidney was sliced and fixed in 4% paraformaldehyde for histology and part of the tissue (cortex and medulla) was stored at −80 °C for further analysis.

The effect of an antioxidant treatment was evaluated in a rat model of metabolic syndrome, Zucker rats. Sixteen female obese Zucker rats were randomly assigned to two groups of 8 rats each. One group received 8 weeks of Mangiferin (Neuron Bio S.A, Granada, Spain), a potent antioxidant, and the other group received placebo. In addition, lean Zucker rats (n = 8) were used as controls. Rats were trained to eat gelatin pellets, which they perceived as a treat. Gelatin pellets were prepared from cooking gelatin (McCormick España SA, Sabadell, Spain) and distilled water using gelatin molds. Two types of pellets were prepared, pellets containing antioxidant and pellets without antioxidant (placebo). The amount of mangiferin within each pellet was adjusted for each rat to provide a dose of 15 mg/kg body weight. Obese Zucker rats received pellets with either mangiferin or placebo and lean rats, pellets with placebo. Rats were fed *ad libitum* and the pellets were administered once daily in the morning during a 8 week period. Control of food intake (g/day) was performed. In addition, body weight was recorded at the beginning and at the end of the experiment.

All experimental protocols were reviewed and approved by the Ethics Committee for Animal Research of the University of Cordoba, and all rats received humane care in compliance with the guiding principles in the Guide for the Care and use of laboratory animals: Eight edition.

### Biochemical measurements in blood and urine

For the experiment with heminephrectomized rats and different content of phosphate diet two days before sacrifice, rats were placed in metabolic cages. Urine samples were collected during a 24 h period for biochemical analysis. At sacrifice plasma ionized calcium was measured using a selective electrode. Creatinine, phosphate and urine protein concentrations were measured using colorimetric assays. Plasma intact parathyroid hormone (PTH) was quantified by Enzyme-Linked Inmuno Sorbent Assay kit (Immutopic, San Clemente, CA). Intact fibroblast growth factor 23 (FGF23) concentration was determined using a FGF23 ELISA kit (Kainos Laboratories, Tokyo, Japan).

In the experiment with Zucker rats, serum levels of creatinine, phosphate, total cholesterol, LDL, HDL, glucose and triglycerides were analyzed using colorimetric assays (Biosystem, Barcelona, Spain). Insulin was evaluated through radioimmunoassay (Millipore, St. Charles, MO, USA).

### Protein isolation and western blot

Cytosolic and nuclear proteins were isolated from renal tissue using specific buffers lisys and the supernatants were centrifuged and stored. In the cytosolic fraction, renal α-klotho protein levels were measured using an anti α-klotho monoclonal antibody (Trans Genic Inc., Kobe, Japan) in a 0.5 mg/ml concentration. β-actin was used as housekeeper. The expression of p65 fragment from nuclear factor light chain kappa B (NF-κB) was determined in the nuclear fraction using polyclonal antibody and transcription factor IIB (TFIIB) expression was determined using a monoclonal antibody (Cell Signaling Technology, Inc., Danvers, MA, USA).

### Histological analysis and immunohistochemistry

Microscopic evaluation of the kidneys was performed in tissue sections (3 μm) fixed with 4% paraformaldehyde solution and embedded in paraffin. Sections were stained with hematoxylin-eosin, Masson Trichromic or periodic acid Schiff. Renal calcification and mineralization was assessed by von Kossa staining. Immunohistochemistry analyses were performed with a Dako Cytomation staining kit (Glostrup, Denmark). Renal sections were dewaxed in xylene, rehydrated in ethanol and incubated at 100 °C in ChemMate™ Target Retrieval Solution pH 6.0 (Dako, Barcelona, Spain) for 20 min. After washing in phosphate buffered saline (PBS), slides were incubated for 10 min in 3% hydrogen peroxide to block endogenous peroxidase. Then, slides were incubated with anti-rat proliferating cell nuclear antigen (PCNA) antibodies (Santa Cruz Biotechnology, Inc.) for 60 min. Subsequently, slides were washed for 5 min in PBS and incubated 30 min with a horseradish peroxidase-labeled polymer. Diaminobenzidine for 5 min was used to develop the brown color of positive areas. Finally, the slides were counterstained with hematoxylin and mounted in Eukitt medium (Labobam, Navarra, Spain).

### Glutathione Peroxidase Activity

At sacrifice, kidney slices were placed in liquid nitrogen to prevent tissue deterioration and subsequently stored at −80 °C. To determine Glutathione Peroxidase (GPx) activity, renal tissue was homogenized and total renal protein was extracted using a specific buffer based on 50 mM Tris-HCl pH 7.5, 5 mM ethylenediaminetetraacetic acid (EDTA), and 1 mM dithiothreitol (DTT). Then, 20 µg of protein was assayed to determine the activity by spectrophotometry with a GPx assay kit (Cayman Chemical Company, Ann Arbor, MI, USA).

### Renal α-klotho expression

Total RNA from kidney tissue was extracted with 1 mL of TRI reagent (Sigma-Aldrich CO). Isolated RNA samples were treated with DNase amplification grade (Sigma-Aldrich CO) following manufacturer instruction. Real-time Polymerase Chain Reaction (PCR) was assayed with 50 ng of treated RNA using SensiFAST SYBR No-ROX One-Step Kit (Bioline Reagents Limited, UK). Primers for PCR were synthesized with Oligo program for α-klotho (Forward 5′-GAAAAT GGCTGGTTTGTCTCG-3′ Reverse 5′-CCTGATGGCTTTTAAGCTTTC-3′) and GAPDH as housekeeping (Forward 5′-AGGGCTGCCTTCTCTTGTGAC-3′ Reverse 5′-TGGGTAGAATCATACTGGAACATGTAG-3′). The expression of target genes was normalized to the expression of GAPDH according to the 2^−ΔΔ^ Ct method.

### Determination of Oxidative Stress *in vitro*

Human Embrionic Kidney-293 (HEK-293) cells were cultured with fetal bovine serum (FBS)-free Dulbecco’s Modified Eagle’s Medium (DMEM) in 96-wells clear bottom black polystyrene microplates. Once cells were at 90–95% confluence, they were supplemented with monobasic and dibasic sodium phosphate in 1:2 proportions to obtain concentrations of 2.6, 3.3 or 5 mM of inorganic phosphate. Other HEK-293 cells were cultured with inorganic phosphate (5 mM) plus melatonin 10^−5^ M that was added 30 minutes before of the phosphate administration. After 24 hours, intracellular content of reactive oxygen species was measured using fluorescent probes 2′,7′-dichlorodihydrofluorescein diacetate (H2DCFDA, Thermo Fisher Scientific, Waltham, MA, USA) at 25 µM, 5-chloromethylfluorescein diacetate (CMFDA, Thermo Fisher Scientific) at 1 µM or Dihydrorhodamine (DHRH, Thermo Fisher Scientific) at 2.5 µM. Probes were incubated at 37 °C for 30 minutes. After incubation, cells were washed and fluorescence was measured with a fluorimeter Tecan F200pro (Tecan Group Ltd, Switzerland) at an excitation/emission wavelength of 485/510 nm.

### Statistical Analysis

Continuous variables are shown as mean (±standard deviation, SD) or median (interquartile range). Categorical variables are presented as percent (%). Baseline characteristics were compared between the two groups, progressors and non progressors using t test or χ^2^ tests as appropriate. Simple correlation analysis was used to compare a relationship between rate of change in eGFR and other variables. Forward stepwise multiple regression analysis was used to identify variables that could predict the change in eGFR. A model to predict progressors vs non–progressor was constructed using logistic regression analysis. Data obtained from experimental work are presented as the mean ± SD; means were compared by one way ANOVA followed by Tukey test as post hoc analysis. All reported P-values were two-sided. A P-value < 0.05 was considered statistically significant. Statistical analyses were performed using SPSS statistical program (SPSS Inc., Chicago, IL, USA).
